# Illness Representations and Quality of Life Among Informal Cancer Caregivers: Examining the Role of Social Support

**DOI:** 10.3390/healthcare14101330

**Published:** 2026-05-13

**Authors:** Chryso Pallari, Eleni Epiphaniou

**Affiliations:** Department of Psychology and Social Sciences, School of Humanities, Social and Education Sciences, European University Cyprus, 2404 Nicosia, Cyprus; chrisopallari@gmail.com

**Keywords:** illness representations, informal cancer caregivers, quality of life

## Abstract

**Highlights:**

**What are the main findings?**

- Caregivers’ therapy control beliefs and emotional representations significantly predicted their mental-health-related quality of life.
- Illness coherence was a significant predictor of physical-health-related quality of life. Social support did not moderate any of these relationships.

**What are the implications of the main findings?**

- The results highlight the need for healthcare professionals to assess and address caregivers’ cognitive and emotional illness representations, as these directly predict caregiver QoL.
- Social support may function more as a general resource rather than a stress buffer, suggesting interventions should not rely solely on support networks but also strengthen caregivers’ understanding of the illness.

**Abstract:**

**Background/Objectives**: The Common-Sense Model (CSM) explains the dynamic framework by which individuals understand and respond to health threats. Research utilizing the CSM highlights the importance of understanding not only patients but also informal caregivers’ cognitive schema regarding the patient’s condition, as these perceptions impact both the patient’s and the caregiver’s physical and mental health. This study examined whether informal cancer caregivers’ illness representations predict their quality of life (QoL) and whether social support moderates these relationships. **Methods**: A cross-sectional online survey was completed by 100 caregivers using validated measures of illness perceptions (IPQ-R), QoL (SF-36), and social support (MSPSS). **Results**: Regression analyses showed that perceived therapy control and emotional representations significantly predicted mental health-related QoL. Illness coherence predicted physical health-related QoL. Moderation analyses indicated that social support did not moderate relationships between illness representations and QoL. **Conclusions**: These findings suggest that caregivers’ cognitive and emotional interpretations of the patient’s illness play an important role in their well-being, whereas social support was not a significant moderator between illness beliefs and QoL related to physical and mental health.

## 1. Introduction

The Common-Sense Model (CSM) explains the dynamic framework by which individuals understand and respond to health threats [1]. This personal framework comprises several dimensions: identity (the label and associated symptoms), causes (attributions regarding the origin of the illness), consequences (perceived threat and impact), timeline (belief about whether the illness is acute or chronic), and control (personal and treatment-related control) [2,3,4]. Subsequent extensions of the model further highlight the role of illness coherence—the extent to which the illness is understood—and emotional representations reflecting affective responses to the condition [5]. Illness perceptions are also shaped by prior experiences and information received from significant others and healthcare professionals [1]. According to the CSM, cognitive and emotional representations guide coping strategies, which in turn influence a range of health outcomes, including physical and mental health, health behaviors, illness management, and quality of life (QoL) [3,6,7,8,9].

Cancer diagnosis is a difficult and often distressing experience that can disrupt many areas of life, require ongoing adjustment, and lead to high levels of psychosocial distress in a substantial proportion of patients [10,11,12]. In the context of rising cancer incidence and increasing strain on healthcare systems, there has been a growing reliance on informal caregivers [13,14]. Family members and close others frequently assume extensive caregiving responsibilities, contributing to medical care management, emotional support, and daily functioning, often with limited formal support [15,16,17,18,19]. While caregiving can be experienced as meaningful, it is also associated with substantial psychological and physical demands [20,21,22].

Research informed by the CSM has demonstrated that caregivers’ cognitive schema regarding the patient’s condition is closely linked to both the patient and the caregiver’s physical and mental health [23,24,25,26]. However, the existing literature has primarily focused on how caregiver illness beliefs influence patient well-being and health-related behaviors [27,28]. Comparatively, fewer studies have investigated how caregiver perceptions of the patient’s illness relate to their own health-related quality of life, physical and psychological health [25,26,29,30], with findings remaining inconsistent.

Social support is a multifaceted concept shaped by social context, access to resources, and both the availability and subjective perception of supportive relationships [31]. Within cancer care, social support is commonly conceptualized as the assistance and encouragement provided by others and has been associated with moderating effects on caregivers’ physical and psychological health [32,33,34]. However, limited research has examined the moderating role of social support in the relationship between caregivers’ illness representations and health outcomes.

Therefore, acknowledging the limited focus on caregiver quality of life within the CSM framework and the potential moderating role of social support, this study explores how caregivers’ perceptions of the patient’s illness predict their own physical and mental health-related quality of life, as well as the extent to which social support moderates these relationships.

## 2. Materials and Methods

### 2.1. Study Design

A cross-sectional survey was conducted using validated questionnaires.

### 2.2. Sample

Informal caregivers of patients with cancer took part in the study. No limitations were implemented in regard to participant age, gender, socioeconomic status, patient’s cancer type and stage. Participants were excluded if the patient was in remission. A total of 100 caregivers were recruited in the study through a combination of purposive and convenience sampling.

### 2.3. Materials

Sociodemographic questions included age, gender (male, female), employment status (whether employed or not), and relationship to the person with cancer (family: spouse/partner, sibling, parent, child; relative: aunt/uncle, cousin, grandparent; other).

The Greek version of the Illness Perception Questionnaire—Revised (IPQ-R) [35] initially developed by Moss-Morris et al. (2002) [5] was used to evaluate caregiver perception of the patient’s illness. Following established practice in the assessment of caregivers’ illness perception [5,24,36], a slightly adapted version was used to measure caregivers’ illness representations. Specifically, items originally framed to assess patients’ illness perceptions were systematically reworded to assess caregivers’ beliefs and interpretations of the patient’s illness. (e.g., ‘I expect him/her to be ill for the rest of their life’, ‘There is a lot I can do to control his/her symptoms.’, ‘What I do can determine whether his/her illness improves or gets worse.’, ‘The course of his/her illness depends on me’, etc.). Dimensions of timeline (Cronbach’s alpha 0.759), cyclical/acute/chronic, treatment control (Cronbach’s alpha 0.850), personal control (Cronbach’s alpha 0.728), consequences (Cronbach’s alpha 0.798), coherence (Cronbach’s alpha 0.826), emotional representations (Cronbach’s alpha 0.882) and causal attributions (Cronbach’s alpha 0.726) of illness were measured. Items are rated on a 5-point Likert scale (1 = strongly disagree to 5 = strongly agree) with subscale scores calculated by summing the relevant items. Items 1, 4, 8, 15, 17, 18, 19, 23, 24, 25, 26, 27, 36 were reversed. Higher scores on treatment control, personal control and coherence indicated more positive beliefs towards the patient’s illness.

The SF-36 Health Survey was used to measure caregiver Qo (Ware, Kosinski, and Gandek, 1993) [37]. It is a 36-item questionnaire that assesses eight dimensions: physical functioning, general health perception, physical role functioning, energy and vitality, bodily pain, emotional role functioning, mental health and social functioning. These dimensions are summarized into two separate scores: the Physical Component Summary (PCS) (Cronbach’s alpha 0.911) and the Mental Component Summary (MCS) (Cronbach’s alpha 0.884). With regard to scoring, the calculation of outcome scores involves transforming the responses for each subscale to a scale ranging from 0 to 100. Following this transformation, the mean score for each subscale is computed based on the rescaled item scores corresponding to that subscale. Higher scores define a more favorable health status.

The Multidimensional Scale Of Perceived Social Support (MSPSS) (Zimet et al., 1988) [38] was used to measure perceived social support from three sources: family, friends and significant others (Cronbach’s alpha 0.955). It is a 12-item questionnaire with items rated on a 7-point Likert scale (1 = ‘very strongly disagree’ to 7 = ‘very strongly agree’). The total score is obtained by summing all item responses, yielding a maximum possible score of 84. Scores ranging from 12 to 35 indicate low perceived social support, scores between 36 and 60 reflect average social support, and scores from 61 to 84 represent high social support.

### 2.4. Data Collection

Data collection commenced after obtaining the required approval from the National Bioethics Committee (No. ΕΕΒΚ ΕΠ 2024.01.358). Sampling was conducted using a combination of convenience and snowball sampling techniques. Convenience sampling was applied, as participants were recruited through established cancer centers. These centers were fully informed about the aims and procedures of the study, and recruitment commenced only after their formal consent was obtained. Following this approval, caregivers were approached by the researcher, screened for eligibility and invited to participate. In addition, snowball sampling was employed, whereby caregivers who had already participated were asked to recommend the study to other individuals within their social network who they believed met the inclusion criteria. Interested individuals contacted the researcher via email and arranged a time convenient for them to meet at the cancer center.

All participants were fully informed about the purpose of the study, the procedures involved, their rights, and issues of anonymity and confidentiality through detailed information sheets. Upon providing informed consent, participants were able to complete the questionnaires via the link included in the information sheet. Completion of the questionnaires (demographic data, IPQ-R, SF-36, MSPSS) took place online, lasted approximately 20 min, and was entirely anonymous. Participants could withdraw from the study at any time using a unique withdrawal code.

### 2.5. Analysis

The statistical analysis was carried out using the IBM SPSS Statistics software, Version 23.0 for iOS. Descriptive statistics and normality tests were implemented first. Multiple regression analysis was used to explore whether caregiver illness beliefs predicted QoL relevant to caregiver physical and mental health. For the moderation analysis, with social support as the moderator, between caregiver illness perceptions and physical and mental health, the SPSS HAYΕS PROCESS model was used.

## 3. Results

Participants’ demographic information appears in Table 1 below. One hundred (N = 100) informal caregivers (age: M = 42.27, SD = 14.856) took part in the study. The majority of participants were women (n = 77), and most participants were employed (n = 87) and cared for their parent (n = 29) or their child (n = 16). The remaining participants cared for their friend, partner, sibling, grandchild, etc. Most of the patients were at stage 4 (n = 39), followed by stage 3 (n = 22) and stage 1 (n = 21).

Considering social support, 84% and 15% of the sample reported receiving high levels and medium levels of social support respectively.

Before conducting multiple regression, Spearman rank correlation was used to examine the relationships between quality of life relevant to physical and mental health and illness representations. The analyses showed weak but statistically significant negative correlations between mental health-related QoL and perceived consequences (r (100) = −0.285, *p* = 0.004) and emotional representations (r (100) = −0.301, *p* = 0.002). Thus, increased perceived consequences of the patient’s illness and negative emotions were related to worse mental health. Results also showed a weak but positive association between control of therapy and QoL relevant to mental health (r (100) = 0.213, *p* = 0.033). The more perceived therapy control, the better the caregiver QoL relevant to mental health.

Subsequent multiple regression analyses with the three dimensions having significant correlations with QoL relevant to mental health were implemented. Results showed that perceived therapy control (B = 1.036, 95% CI (.071, 2.000), *p* = 0.036) and emotional representations (B = −0.987, 95% CI (−1.956, −0.019), *p* = 0.046) were the most important predictors for QoL relevant to mental health, jointly explaining the 14% (adjusted R^2^) of its variance. Higher treatment control predicted better QoL relevant to mental health, whereas negative emotional representations predicted poorer QoL relevant to mental health, with the former being the stronger predictor.

Spearman rank correlation was then used to explore the relationship between illness representations and physical health-related QoL. Results showed a weak but statistically significant negative association between QoL relevant to physical health and immunity, r (100) = −0.266, *p* = 0.007, and emotional representations, r (100) = −0.212, *p* = 0.034. Additionally, there was a weak but statistically significant positive association between QoL relevant to physical health and coherence, *p* < 0.05, indicating that greater understanding of the illness was associated with better physical health-related QoL.

Subsequent multiple regression analyses, including the three illness representation dimensions having significant correlations with QoL relevant to physical health, were conducted. The overall model was statistically significant (F(3) = 4.626, *p* = 0.005) and accounted for 10% of the variance in physical health-related quality of life (adjusted R^2^). Illness coherence emerged as the only significant predictor, with higher coherence associated with better physical health-related QoL (B = 1.085, 95% CI [0.065, 2.104], *p* = 0.037).

Moderation analyses were then performed to examine whether perceived social support moderated the relationships between treatment control and mental health-related QoL, as well as between emotional representations and mental health-related QoL. The interaction effects between perceived treatment control and social support (b = −0.253, SE = 1.178, t(100) = −0.215, *p* = 0.830) and between emotional representations and social support (b = 0.847, SE = 0.937, t(100) = 0.904, *p* = 0.368) were not statistically significant, indicating no moderating effect. Similarly, social support did not moderate the association between illness coherence and physical health-related QoL (b = −1.302, SE = 1.2037, t(100) = −1.080, *p* = 0.2828).

## 4. Discussion

The present study examined whether caregivers’ illness representations related to the patient’s cancer predicted caregivers’ quality of life in terms of physical and mental health, and whether perceived social support moderated these associations. The findings indicated that perceived treatment control and emotional representations were significant predictors of mental health-related QoL, with emotional representations emerging as the stronger predictor. Illness coherence was identified as the only significant predictor of physical health-related QoL. Social support did not exert a moderating effect on the relationships between illness representations and either physical or mental health-related quality of life.

The patterns that caregivers’ negative emotional representations and perceived treatment control are significant predictors of mental health-related quality of life, whereas coherence was a significant predictor of physical health-related QoL are consistent with previous research [39,40,41] and suggest that better mental health-related quality of life is related to grater treatment control and fewer negative emotional representations to the patient’s illness while better confidence and ability to understand illness predicted better physical health-related QoL.

Contrary to other studies, additional illness representations such as causal attributions and perceptions of illness timeline did not emerge as significant predictors for caregiver mental and physical health [26,42,43,44]. The sample comprised caregivers of patients with diverse cancer diagnoses, at varying stages of the disease, and with differing relational ties to the patient. This heterogeneity may explain why some illness representations did not emerge as significant predictors. This discrepancy might reflect variation in illness perceptions among caregivers who support patients with different cancer types and at different stages of the disease [45].

Furthermore, variability in findings regarding caregiver illness perceptions might be attributable to differences in caregiver intensity and caregiver involvement [42], as well as to the different measurement approaches [43]. Taken together, these differences suggest that inconsistencies across studies may be partly explained by differences in caregiver populations—particularly with respect to the illnesses of the care recipients—as well as by variation in the instruments used to capture the complexity and multidimensionality of illness perceptions [3,24,46].

A substantial body of research shows that social support plays an important buffering role for caregivers’ mental and physical well-being [47,48], with some studies identifying its moderating role on caregivers’ mental health [49,50]. The absence of the moderating role of social support in the current study between illness cognitions and QoL relevant to physical and mental health indicates that social support may not influence the direction of these relationships in this specific sample, as most of the caregivers in the current study reported receiving high levels of social support, resulting in limited variability in the moderator. This lack of variability might have reduced the possibility of detecting interaction effects. Regarding the moderation analysis, it is important to note that statistical power is often reduced in moderation analyses due to interaction and separate conditional models. In the present study, as the moderator variable was skewed and the study sample was small, certain regions of its distribution may have been underrepresented, which can further decrease statistical power and possibly moderation effects.

It is also possible that moderation was not significant because the stress-buffering effect of social support emerges only under increased stress levels, elevated caregiver burden, and vulnerability, factors not tested in the current study. Thus, it is more likely to have a moderating effect when caregivers experience severe stressors.

The present study has several limitations. Its cross-sectional design prevents conclusions about causality and does not allow for the observation of changes over time. In addition, participants cared for patients with different types of cancer and at different stages of the disease, which may be associated with varying illness representations and social support needs. Lastly, convenience sampling was used, which may limit the study’s external validity and the applicability of the findings to a broader population [51].

## 5. Conclusions

The findings from the present study support the importance of informal caregiver perceptions of treatment control and emotional representations in relation to mental-health QoL, as well as the role of illness coherence and understanding in QoL relevant to physical health. These cognitive schemas warrant attention from healthcare professionals, as they seem to predict caregivers’ well-being. Accordingly, interventions for this population may focus on providing a clear explanation about the illness, aiming to enhance caregivers’ understanding and perceptions of treatment control, and thereby reduce negative emotional responses associated with the illness. Lastly, further research is needed to examine the relationship between caregiver illness beliefs, mental and physical well-being and identify patterns and consistencies across caregiver populations caring for patients with different illnesses.

## Figures and Tables

**Table 1:** Participant demographic characteristics (N = 100).

Characteristic		n (%)
Gender	Female	77
	Male	23
Employment	Employed	87
	Unemployed	13
Relationship with the patient	Parent	29
	Child	16
	Grandparent	13
	Partner	10
	Uncle	9
	Sibling	8
	Friend	6
	Other	9
Cancer stage	Stage 4	39
	Stage 3	22
	Stage 1	21
	Stage 2	17

## Data Availability

The data presented in this study are available on request from the corresponding author. The data are not publicly available due to privacy or ethical restrictions.
